# TTFDNet: Precise Depth Estimation from Single-Frame Fringe Patterns

**DOI:** 10.3390/s24144733

**Published:** 2024-07-21

**Authors:** Yi Cai, Mingyu Guo, Congying Wang, Xiaowei Lu, Xuanke Zeng, Yiling Sun, Yuexia Ai, Shixiang Xu, Jingzhen Li

**Affiliations:** Optoelectronic Devices and Systems of Ministry of Education and Guangdong Province, Shenzhen Key Lab of Micro-Nano Photonic Information Technology, State Key Laboratory of Radio Frequency Heterogeneous Integration, College of Physics and Optoelectronic Engineering, Shenzhen University, Shenzhen 518060, China

**Keywords:** fringe projection profilometry, depth estimation, deep learning, transfer learning

## Abstract

This work presents TTFDNet, a transformer-based and transfer learning network for end-to-end depth estimation from single-frame fringe patterns in fringe projection profilometry. TTFDNet features a precise contour and coarse depth (PCCD) pre-processor, a global multi-dimensional fusion (GMDF) module and a progressive depth extractor (PDE). It utilizes transfer learning through fringe structure consistency evaluation (FSCE) to leverage the transformer’s benefits even on a small dataset. Tested on 208 scenes, the model achieved a mean absolute error (MAE) of 0.00372 mm, outperforming Unet (0.03458 mm) models, PDE (0.01063 mm) and PCTNet (0.00518 mm). It demonstrated precise measurement capabilities with deviations of ~90 μm for a 25.4 mm radius ball and ~6 μm for a 20 mm thick metal part. Additionally, TTFDNet showed excellent generalization and robustness in dynamic reconstruction and varied imaging conditions, making it appropriate for practical applications in manufacturing, automation and computer vision.

## 1. Introduction

Fringe projection profilometry (FPP) is a computer vision method that uses a projector to cast a known pattern onto an object’s surface. It captures images of the deformed pattern with a camera to extract depth information and reconstruct the object’s three-dimensional shape. As reported, it can be used to enhance some functionalities like navigation, object manipulation, augmented reality and facial recognition [1,2,3,4]. Due to its non-contact attributes and straightforward equipment requirements, FPP has found applications across diverse fields, such as robotics, medical imaging, industrial automation and consumer electronics [5,6,7]. In FPP, phase retrieval is one of the critical steps, which is traditionally achieved by some phase reconstructions including Fourier transform profilometry (FTP) [8,9,10] and some of its variants like windowed Fourier transform profilometry (WFT) [11] and wavelet transform (WT) [12]. All of them work on modulating high-frequency fringes on the object surface to separate the target information from the background intensity. However, the complexity of surface depths and edges can lead to reconstruction failure due to spectral aliasing [13]. Phase shift profilometry (PSP) [14,15,16] could obtain a wrapped phase by recording three or more phase-shifted sinusoidal fringe patterns and then unwrapping them to further reconstruct objects with complex surfaces [17]. During phase unwrapping, spatial phase unwrapping (SPU) and temporal phase unwrapping (TPU) are commonly used. SPU uses phase differences between adjacent pixels for unwrapping, such as rhombus type [18] and curtain type [19]. In contrast to SPU, which depends on neighboring phase values, TPU algorithms obtain the absolute phase for each point by introducing extra patterns. Among these, the Gray code algorithm [20] and the multi-heterodyne algorithm [21] are widely used, while advanced methods, such as fringe amplitude encoding [22], are also being actively researched. Based on TPU methods, phase-differencing profilometry (PDP) [23] leverages number-theoretical TPU (NT-TPU) [24] to compute phase-shifting deformed patterns, achieving high computational efficiency and strong algorithmic robustness for high-speed 3D measurement. Similarly, the phase-shifting temporal phase unwrapping (PS-TPU) [25] algorithm incorporates phase-shift coding [26], which achieves high-speed, accurate 3D measurements with fewer projections. This approach offers improved robustness to reflectivity changes and outperforms traditional TPU methods like the Gray code [20] and phase coding [27]. Also based on phase shifting, unlike PS-TPU, which collects three frames of deformed patterns based on phase shifting, modulation measuring profilometry (MMP) [28] achieves high-speed, dynamic 3D measurement with a single shot. Single-shot methods simplify system design and synchronization, offering efficiency and suitability for many dynamic applications. One such method is computer-generated Moiré profilometry (CGMP) [29]. This technique differs from traditional Moiré profilometry (MP) [30] by using computer-generated fringes and a single-shot approach, which simplifies the equipment and enhances its suitability for real-time tasks. Consequently, many efforts have been made to realize phase retrieval based on a single fringe pattern [31,32,33,34] in FPP.

In recent years, deep neural networks (DNNs) have been applied to predict single-frame fringe patterns in FPP. Instead of the traditional multi-frame records, DNNs only need a one-time pre-train. Compared with the traditional methods, DNNs can automatically recognize subtle features in fringe patterns more effectively, thereby improving the accuracy and speed. Many methods have been reported, such as PhaseNet [35] and PhaseNet 2.0 [36], using neural networks to predict fringe order and improve phase unwrapping accuracy. Others involve predicting the numerator and denominator of the wrapped phase or using composite fringes for phase retrieval [37,38,39,40]. It is worth noting that all the methods above work only as an intermediate step to extract the depth information; therefore, the deviations from phase information may be exaggerated and thus degrade spatial resolution or accuracy in the subsequent calculations required to obtain depth information. Accordingly, directly predicting the depth map from single fringe patterns becomes appealing. Recently, convolutional neural network (CNN) models have been proposed for the end-to-end depth estimation from single-frame fringe patterns to depth maps [41,42,43,44]. However, CNNs face challenges in accurately capturing the global structural intricacies and local fine details of complex surfaces. This limitation often leads to a loss in spatial resolution or accuracy in the reconstruction of depth information. Given the success of transformer models in handling global dependencies in depth estimation for indoor and outdoor scenes using large datasets [45,46,47], transformer-based approaches for FPP have been proposed for direct prediction from single-frame fringe patterns to depth maps, such as SwinConvUNet [48] and PCTNet [49]. However, the effectiveness of transformers heavily relies on large datasets. Consequently, it remains challenging to fully leverage transformers’ capabilities in data-limited FPP tasks, resulting in underutilization of their potential and unnecessary computational overhead.

To address these issues, we adopt a more feasible approach in our work by fine-tuning and modifying the network architecture based on a pre-trained ViT model. Subsequently, we tailor a novel loss function to facilitate the transfer of general knowledge from the pre-trained model to FPP, ensuring effective performance even on small datasets. Specifically, the proposed TTFDNet model is structured with three key components: the precise contour and coarse depth (PCCD) pre-processor, the global multi-dimensional fusion (GMDF) module and the progressive depth extractor (PDE). The PCCD module leverages transfer learning to enhance feature extraction, which is then fused with original features in the GMDF module. These fused features are then processed by the PDE to achieve precise depth estimation. Additionally, to further improve fine-tuning and training, we design and implement a fringe structure consistency evaluation (FSCE) based on FPP as a loss function. Experiments show that TTFDNet achieves high prediction accuracy with minimal measurement deviation: 0.024% for plane accuracy and 0.354% for spheroids. The proposed model is suitable for fast depth estimation from single-frame fringe patterns and real-time 3D reconstruction.

## 2. Materials and Methods

**Figure 1:**
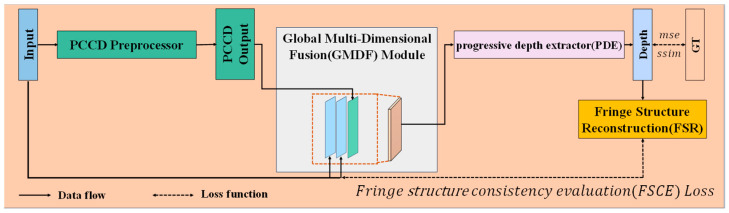
Overview of the TTFDNet model.

In this study, we introduce a network for depth estimation from single-frame fringe patterns. The overall structure is illustrated in Figure 1.

First, the input is passed through the PCCD pre-processor, which extracts precise contour information and coarse depth features. Next, the output from the PCCD pre-processor is then fused with the original input in the GMDF module, creating tri-channel data with precise contour information, coarse depth features and original features. Then, the fused data are processed by the PDE to generate the final depth map. During the training process, the depth map is compared using a composite loss function, which consists of two components. These comparisons help in optimizing the backpropagation and improving the model’s performance. In the following sections, we will provide a detailed description of each module in this network. 

The inputs are deformed fringe patterns obtained from the CCD and can be represented as

(1) $$I\left(x,y\right)=A\left(x,y\right)+B\left(x,y\right)\cos\left[\varphi_{0}\left(x,y\right)+∆\varphi \left(x,y\right)\right],$$

where $\varphi_{0}$ is the initial phase distribution of the projected fringes, and $∆\varphi \left(x,y\right)$ is the phase modulation resulting from the object’s surface depth distribution $D\left(x,y\right)$, which can be expressed by $∆\varphi \left(x,y\right)=2\pi fD\left(x,y\right)d/l.$ Here, f is the fringe frequency; d is the distance between the optical centers of the projector and the camera; and l is the distance from the camera to the object.$A\left(x,y\right)\;$and $B\left(x,y\right)$ represent the background gray value and the modulation intensity of the fringes, respectively. A smaller $B\left(x,y\right)\;$value indicates a less deformed fringe, which may belong to the background. In contrast, a larger $B\left(x,y\right)\;$value suggests the object region in an image, differentiating from the background. Typically, a fixed threshold for $B\left(x,y\right)$ is used to distinguish object and background regions, which can result in unstable predictions and limited generalization due to scene inconsistencies. To improve the stability and adaptability, we introduce a PCCD pre-processor based on a vision transformer (ViT) pre-trained model [50] originally developed for image recognition. This model enables the neural network to automatically identify fringes with minimal or no modulation, thereby accurately distinguishing the object region from the background.

**Figure 2:**
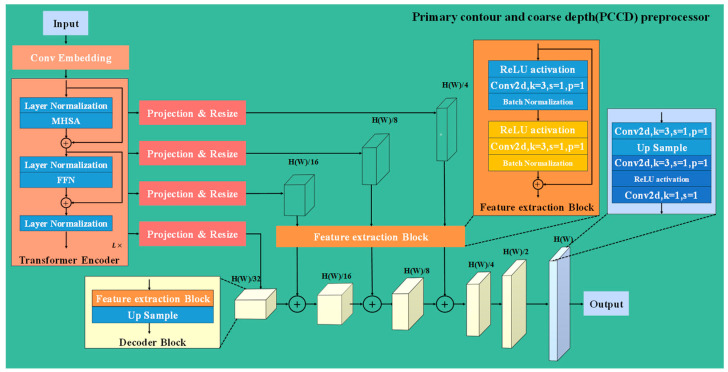
The schematic diagram of precise contour and coarse depth (PCCD) pre-processor.

The PCCD architecture utilizes 4 pre-trained transformer encoder layers for feature extraction, as shown in Figure 2, integrated within a sophisticated decoding process. In PCCD, the input data first undergo embedding and layer normalization to mitigate internal covariate shift and promote faster convergence. Then, the multi-head self-attention (MHSA) mechanism identifies and emphasizes features from different parts of the patterns, enabling the model to adaptively focus on various spatial regions. After attention processing, the data are normalized again and passed through a feed-forward network (FFN) with two linear transformations and a Gaussian error linear unit (GeLU) activation. Additionally, residual connections around both the MHSA and FFN modules preserve information integrity and prevent gradient vanishing in deeper layers. Decoding starts from the deepest encoder layer and proceeds through a decoder block, which includes a feature extraction block with convolutional layers, ReLU activation and batch normalization, followed by up-sampling. Subsequent layers are refined through the feature extraction block after projection and resize, and their outputs are fused with previously processed features. This fused output undergoes further refinement and up-sampling by the decoder block, enhancing the resolution and detail for a more accurate prediction. This iterative strategy allows for progressive refinement of depth estimates.

**Figure 3:**
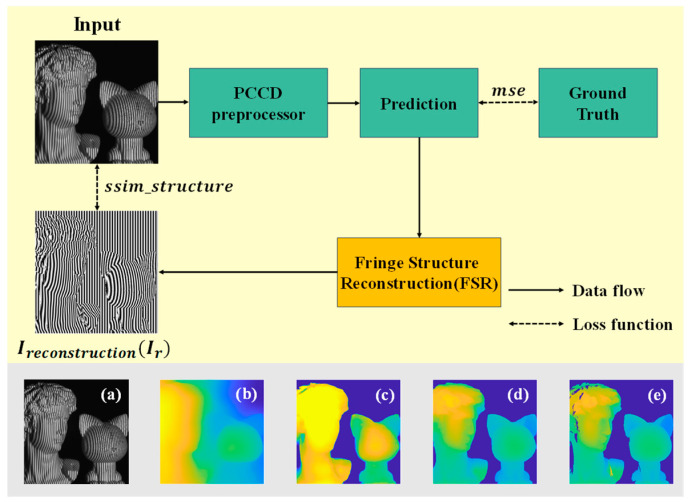
FSCE process for fine-tuning PCCD pre-processor. (**a**) Input fringe pattern. (**b**) PCCD prediction before any fine-tuning. (**c**) PCCD prediction after fine-tuning without FSCE. (**d**) PCCD prediction after fine-tuning with FSCE. (**e**) Ground truth.

Since PCCD is built upon a pre-trained vision transformer (ViT) model [50], we fine-tune it for FPP. To achieve this, we augment it with fringe structure consistency evaluation (FSCE) and integrate this into the loss function, as illustrated in Figure 3.

Equation (1) describes how depth distribution on an object’s surface modulates fringe patterns, enabling fringe structure reconstruction (FSR) from depth prediction to obtain $I_{restruction}(I_{r})$. Since fringe deformation is primarily influenced by surface depth distribution, the key differences lie in the structural variations between $I_{r}$ and the input. Therefore, we fine-tune the model on the fringe projection dataset using a composite loss function. This loss function integrates the structural component of SSIM for capturing essential structural changes between $I_{r}\;$and the input, along with the mean squared error (MSE) for depth prediction and ground truth (GT). It can be expressed as

(2) $$L=\lambda_{1}\cdot mse\left(pred,GT\right)+\lambda_{2}\cdot ssim\left(FSR(pred),input\right),$$

where $\lambda_{1}$ and $\lambda_{2}$ are the weight coefficients, and $\lambda_{1}+\lambda_{2}=1$. These coefficients balance the contributions of the mean squared error (MSE) and the structural similarity index measure (SSIM). Through extensive experimentation, we found that setting $\lambda_{1}=0.7$ and $\lambda_{2}=0.3$ provides a good trade-off between minimizing pixel-wise errors and preserving structural details. By utilizing the composite loss function and conducting fine-tuning on the pre-trained model, it becomes possible to transfer extensive general knowledge acquired from image recognition tasks (e.g., understanding spatial relationships and object textures) to fringe projection depth prediction across diverse scenes. In Figure 3, the gray section at the bottom illustrates the process where the input (Figure 3a) undergoes pre-processing in the PCCD system. Before any fine-tuning, the depth estimation in (b) shows significant inaccuracies in both depth and object contours. After fine-tuning, the results improve, as seen in (c) and (d). Figure 3c shows the outcome without the inclusion of fringe structure consistency evaluation (FSCE), while Figure 3d includes FSCE. As a result, (d) is much closer to the ground truth (GT) shown in (e), with more precise contours and clearer details. This demonstrates that incorporating FSCE enhances the model’s ability to capture fine variations in depth.

The output from the fine-tuned PCCD contains global information about precise contours and coarse depth. This output is then fed into the GMDF module, which forms a residual connection with the original input, ensuring the preservation of initial information. The fused data from the GMDF module are processed by the PDE for further depth feature extraction. Additionally, both the PDE and PCCD modules employ residual connections with intermediate features. Together with the original input residual connection in the GMDF, this creates a dual residual structure that retains crucial information from both the original input and the intermediate stages. This structure enhances depth feature extraction and overall performance.

**Figure 4:**
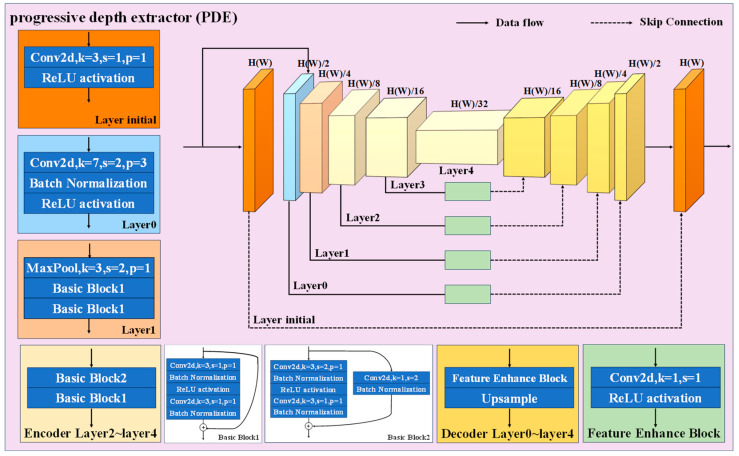
The structure of progressive depth extractor (PDE).

As the final module of depth feature extraction, the PDE is highlighted in Figure 4, with a U-shaped architecture enhanced by the ResNet-18 encoder, designed to improve deep feature extraction and precision in spatial reconstruction. At the beginning, the input data are simultaneously fed into both the ‘Layer initial’ and ‘Layer0’, establishing a dual path. In the ‘Layer initial’, the input undergoes convolution and ReLU activation to preserve the original image features. These features are then enhanced through skip connections, ensuring detail fidelity throughout the network. Meanwhile, in the encoder, the input is processed through five layers (Layer0 to Layer4). Layer0 uses a 7 × 7 convolutional kernel with a stride of 2 and padding of 3, allowing for extensive spatial information extraction and a broader contextual scope. The subsequent layers intensify feature extraction using down-sampling and basic blocks, which consist of sequences of convolutions, batch normalization and activations. Each layer’s output undergoes dimension reduction and channel enhancement and passes through a feature enhancement block, which links to the corresponding decoder layer via skip connections. In the decoder, the upscaled output from each preceding layer is merged with the skip-connected output from the corresponding encoder layer. These integrated data are then processed through convolution and up-sampling, expanding spatial dimensions and reducing channels in the feature maps. At last, the output from the PDE is compared with the ground truth (GT) using MSE loss, then reconstructed by the FSR and evaluated using FSCE against the input, as described in Equation (2). This process is used to train the model.

To sum up, the depicted architecture demonstrates an advanced model using dual residual mechanism and knowledge transfer to enhance depth estimation accuracy. The proposed TTFDNet consists of three main modules: the PCCD pre-processor, the GMDF module and the progressive depth extractor (PDE). The PCCD pre-processor, built and fine-tuned from ViT pre-trained model, extracts precise contour and coarse depth features. This extraction is integrated with the original input through the GMDF module. The integrated output from the GMDF module is fed into the PDE, which employs a U-shaped architecture enhanced by a ResNet-18 encoder, further refining these features to produce accurate depth estimations. To improve training and tailor the model to fringe projection, FSCE is designed to form a composite loss function. This composite loss enhances training effectiveness and ensures the transfer of generalized pre-trained knowledge to specific FPP physical knowledge.

## 3. Results and Discussion

### 3.1. Establishing a Dataset

To establish a dataset for training and evaluation of the proposed TTFDNet model, we developed a sophisticated projection-camera system. This system comprised an Anhua M11B (LC)-DEMO projector (Shenzhen Anhua Optoelectronics Technology Co., Ltd., Shenzhen, China), featuring a DMD resolution of 1920 × 1080, and a BASLER acA2440-20 gm camera equipped with a 12 mm focal length lens. And the camera’s optical axis was maintained perpendicular to the object plane at a distance of approximately 0.5 m. 

**Figure 5:**
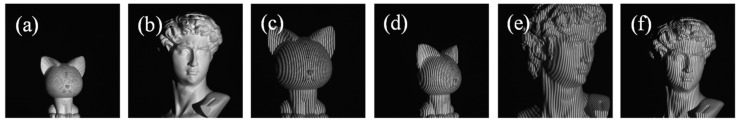
Objects (**a**,**b**) and fringes projected onto objects (**c**–**f**).

Fringe patterns with an 8-pixel width were projected onto a variety of targets, including plaster statues of David (8 cm wide, 14 cm high) and cat-shaped (5 cm wide, 10 cm high) ceramics, as shown in Figure 5a,b. To generate a diverse and comprehensive dataset, these objects were randomly rotated, capturing deformed fringe images from multiple angles, as shown in Figure 5c–f. These patterns serve as inputs for the TTFDNet model. The ground truth maps are obtained through a specific process: initially, the four-step phase shifting [51] and phase unwrapping algorithm [21] are used to derive the unwrapped phase. Subsequently, the unwrapped phase is converted to depth values through phase-to-depth mapping. This involves using five reference planes with known distances to project fringe patterns and capture corresponding unwrapped phase values. By fitting a second-order polynomial to these measurements, an accurate phase-to-depth relationship is established, ensuring precise conversion of unwrapped phase data to depth measurements and thus providing the essential ground truth.

The resulting dataset comprised 1062 samples, each consisting of a 384 × 384 PNG image as input and a corresponding MAT format matrix of the same resolution as the ground truth (GT). This dataset was systematically divided into three subsets: 646 samples for training, 208 samples for validation and 208 samples for testing. For network training, we utilized a computational setup featuring a 30GB RAM CPU and two T4 GPUs, employing the Adam optimizer with a learning rate of 1 ×10^−5^. With a batch size of 4, optimal model performance was attained after 500 epochs.

### 3.2. Qualitative and Quantitative Results of Static Targets

**Figure 6:**
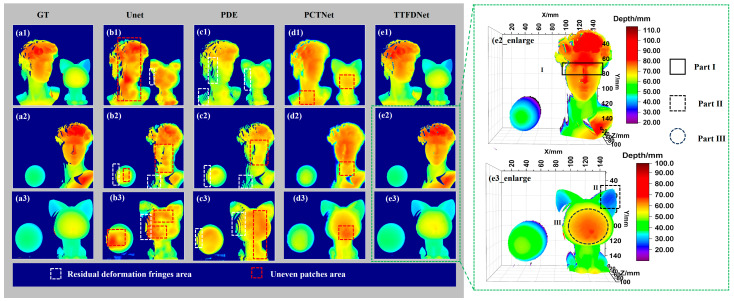
Comparison of depth prediction and 3D reconstruction using the proposed model.

We conducted experiments to validate the efficacy of the proposed model by comparing the depth prediction results using four methods: Unet, PDE, PCTNet [49] and TTFDNet. All four models were trained on the same training dataset and tested on the same test set. The gray section in Figure 6 shows the depth predictions using various methods. 

The gray sections represent depth maps, the five columns (left to right)) show ground truth and predictions from four methods, and the three rows (top to bottom) display different combination scenarios. The green dashed box shows 3D reconstructions by the proposed method. Depth predictions based on Unet and PDE exhibit noticeable errors, with distinct colors indicating significant depth disparities. Specifically, the details of David’s face in Figure 6(b1,c1,b2,c2), as well as the cat’s face in (b3,c3), are inadequate, showing uneven patches (highlighted by red dotted frames). Additionally, there are residual deformation fringes (highlighted by white dotted frames) present caused by high-frequency fringes that capture minute surface variations but impose greater demands on the neural network. Integrating the transformer architecture significantly improves performance. Although PCTNet’s predictions surpass those of pure CNN networks, they still fall short in capturing local details and exhibit uneven patches (as indicated by red dotted frames in Figure 6(d1–d3)). In contrast, TTFDNet demonstrates the highest accuracy and consistency with the ground truth (GT) in Figure 6(a1–a3). It effectively eliminates residual deformation fringes (white dotted frames) and uneven patches (red dotted frames), excelling in preserving intricate object details. This evidence underscores the suitability of the proposed TTFDNet model for FPP tasks, particularly when dealing with limited datasets. Enlarged views in Figure 6, showcasing the 3D reconstruction results based on the proposed network, further highlight its ability to accurately capture the fine details. These include eye contours and intricate eyeball features (Part I), subtle notches in the cat’s ears (Part II) and finer facial details (Part III).

**Figure 7:**
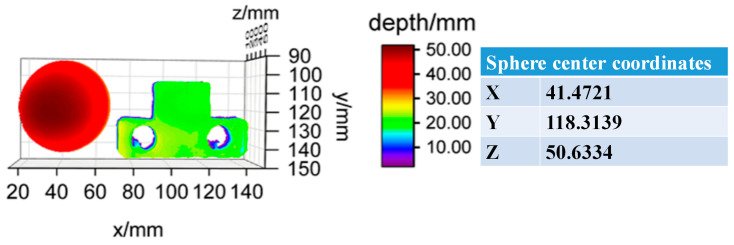
A 3D reconstruction of standard parts based on TTFDNet.

Based on the qualitative advantages demonstrated by the proposed model, we further validated the accuracy of TTFDNet through comprehensive quantitative assessments. We first calculated the mean absolute error (MAE) on a test set of 208 scenes. The Unet, PDE, PCTNet and TTFDNet models achieved MAE values of 0.03458 mm, 0.01063 mm, 0.00518 mm and 0.00372 mm, respectively. Notably, the proposed TTFDNet model exhibited a MAE nearly 10 times lower than that of Unet, underscoring its superior performance for single-frame FPP depth prediction. Additionally, we performed another quantitative evaluation using standard parts, including a sphere and a metal workpiece. We set up a zero-reference plane and placed the spherical and planar standards in front of the reference plane. Depth measurements were then taken from the surface of the standard parts to the reference plane. The metal workpiece has a standardized thickness of 20.0000 mm, and the standard sphere has a radius of 25.4000 mm. Figure 7 depicts the 3D reconstruction of these standard parts based on depth predictions from the TTFDNet model. For the spherical standard part, the reconstructed sphere was fitted using least squares, resulting in center coordinates of (41.4721, 118.3139, 25.1429). The Z value at these coordinates was calculated to be 50.6334 mm, and the predicted radius was approximately 25.4905 mm. By taking the difference between the predicted values and the actual value of the standard sphere as the deviation, the proposed model represents a radius prediction deviation of about 90.5 μm, with a deviation rate of approximately 0.354%. 

Table 1 provides detailed quantitative results for both the sphere and the metal workpiece. The depth value was calculated as the distance from the standard part to the zero-reference plane. Points with an error greater than 10 μm were identified as incompatible and excluded, resulting in 99.6% valid points. The average value of these valid points was then computed as the predicted thickness. The plane accuracy prediction for the metal workpiece showed a deviation of approximately 6.2 μm, corresponding to a deviation rate of about 0.024%. These findings highlight the accurate depth map prediction capabilities of TTFDNet from single-frame fringe patterns captured by a monocular camera. Overall, these quantitative evaluations confirm the robust and precise predictive capabilities of the TTFDNet model.

### 3.3. TTFDNet Applied to Dynamic Scene

**Figure 8:**
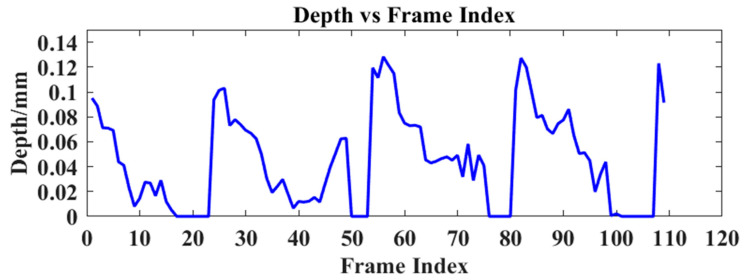
The height of a certain point on the fan changes.

Following the qualitative and quantitative analyses, we further validated the TTFDNet model’s performance in dynamic scenarios. This experiment involved capturing the image sequences of a fan with a diameter of approximately 10 cm and four blades, rotating at 5 revolutions per minute from various angles and positions. The model was tasked with predicting the depth values and tracking the changes in a specific fan point over time. We randomly selected a coordinate point that the fan blade will pass through and recorded the depth of this point for each frame of the predicted depth map. As depicted in Figure 8, the depth variations of this point are plotted across multiple frames. The predicted depth results demonstrate the model’s ability to accurately capture subtle depth fluctuations on a frame-by-frame basis, with periodic depth value shifts corresponding to the fan blade passing the selected point. Additionally, this method is effective in identifying slight changes caused by vibrations, within the range of 0–0.12 mm.

**Figure 9:**
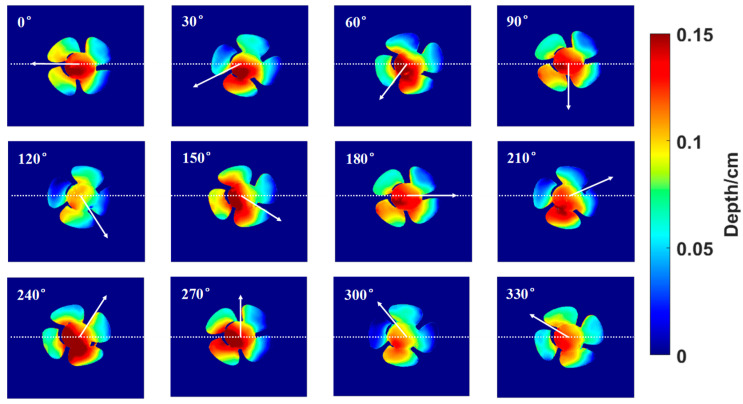
The predicted depth maps for a rotating fan.

To further illustrate the model’s performance in dynamic scenarios, Figure 9 displays a series of predicted depth maps for the rotating fan, taken at 30-degree intervals. These maps capture the depth variation of the fan blades during counterclockwise rotation, with color variations from blue (low) to red (high) indicating depth changes. The consistent depth predictions across these frames confirm the model’s ability to accurately track the shape and position changes of the fan throughout its rotation.

### 3.4. Robustness and Generalization Capabilities of TTFDNet

**Figure 10:**
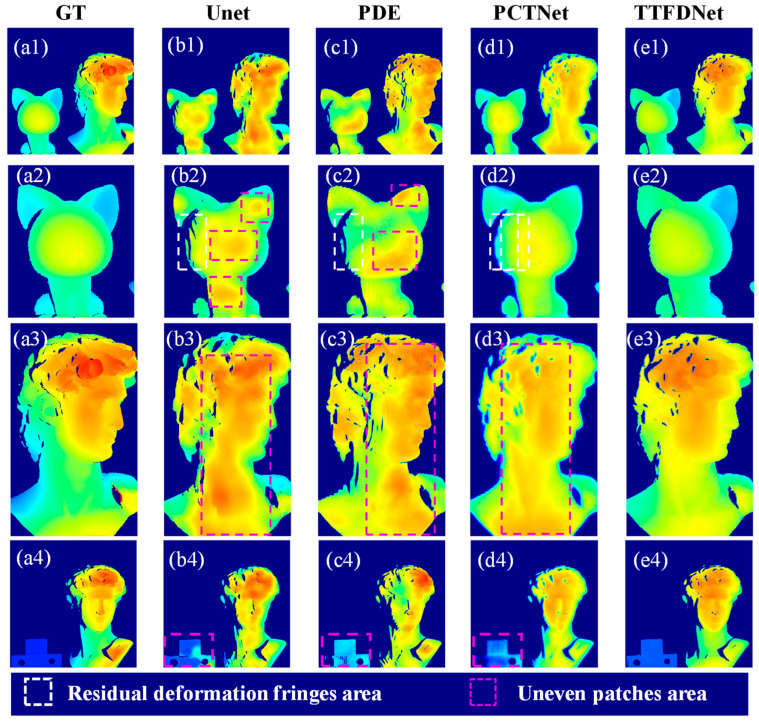
Predicted depth maps in varied imaging conditions. From left to right are the ground truth and predictions from four different methods. (**a1**–**e1**) show the overall scene; (**a2**–**e2**) are zoomed-in views of the left object from the overall scene; (**a3**–**e3**) are zoomed-in views of the right object from the overall scene; (**a4**–**e4**) show the predicted maps of scenes composed of different objects.

The conventional method of FPP depth prediction is often sensitive to changes in projection and imaging system parameters, which can result in distorted depth estimations. To evaluate the model’s robustness, we introduced variations to the system parameters. We shifted the position of the projection system 1 cm horizontally while simultaneously lowering the position of the imaging system 1 cm vertically. Additionally, the focus was adjusted by rotating the focus ring on the lens to achieve clear images. Subsequently, we collected new deformed fringe patterns under these altered conditions to assess the model’s performance. The prediction results are presented in Figure 10, with the second and third rows providing enlarged views of two objects from the first row. These enlarged views offer a detailed examination of the model’s ability to handle deformations, demonstrating its robustness against changes in system parameters.

In comparing the results, it is evident that the Unet, PDE and PCTNet models struggle with detail retention and clarity, with residual deformations in the white frames of Figure 10(b2,c2) and uneven depth predictions in the pink frames of Figure 10(b2,c2,b3,c3,d3,b4,c4,d4). In contrast, the TTFDNet model demonstrates superior performance, avoiding deformation fringes and preventing uneven patches, as shown in column e. These results highlight the TTFDNet model’s exceptional robustness and reliability in handling variations in system parameters, maintaining high-quality depth predictions even under challenging conditions.

Despite these strengths, several limitations should be considered. The TTFDNet model employs multi-dimensional information fusion and a pre-trained vision transformer, which require significant computational resources and time for training and inference. To address this, future research could focus on optimizing the model architecture to enhance efficiency. For instance, using knowledge distillation, a lightweight CNN can be designed as the student model, with the pre-trained large model serving as the teacher model. This approach may maintain performance while reducing computational demands. Furthermore, while the model demonstrates robustness to system parameter variations within a certain range, its performance may still be affected under extreme conditions, such as significant displacements or large aperture changes. To mitigate this, future work could explore dynamically adjusting model parameters or incorporating adaptive algorithms to enhance the model’s performance under these extreme conditions.

## 4. Conclusions

This study introduces the TTFDNet model for depth estimation from single-frame fringe patterns. The proposed model integrates several key components, including a PCCD pre-processor for preliminary depth extraction, a GMDF module for multi-dimensional information fusion and a PDE module for precise depth extraction. Additionally, fringe structure consistency evaluation is employed to facilitate the transfer of general knowledge from a pre-trained vision transformer (ViT) model to the domain of fringe projection profilometry (FPP), enhancing the overall network training process. In a comprehensive evaluation on a test set of 208 scenes, TTFDNet achieved an average mean absolute error (MAE) of 0.00372 mm. This performance significantly surpasses that of the Unet model (0.03458 mm), PDE model (0.01063 mm) and the existing end-to-end transformer-based model PCTNet (0.00518 mm). These results underscore the superiority of TTFDNet, particularly in leveraging transfer learning to harness the capabilities of transformers for FPP depth estimation, even with a limited dataset. Further validation using standard parts confirms the precision of TTFDNet, with deviations as low as approximately 90 µm for a 25.40 mm radius sphere and approximately 6 µm for a 20.00 mm thick metal workpiece. Both qualitative and quantitative analyses validate the model’s superiority in depth estimation. Dynamic reconstruction experiments involving a rotating fan, as well as robustness validation under varying imaging conditions, further attest to the model’s effectiveness. TTFDNet consistently demonstrated reliable performance in dynamic scenarios and robust generalization across different imaging conditions. 

In conclusion, the reliable performance of TTFDNet in both dynamic and static conditions, coupled with its robust generalization capabilities, makes it highly suitable for practical applications in fields such as manufacturing, robotics and computer vision. 

## Figures and Tables

**Table 1:** Quantitative results of standard workpiece.

	Thickness	Radius
Standard value (mm)	20.0000	25.4000
Predictive value (mm)	19.9938	25.4905
Deviation (μm)	6.2	90.5

## Data Availability

The data supporting the findings of this study are available upon reasonable request from the corresponding author.

## Abbreviations

**Acronyms**	**Full Forms**
FPP	Fringe Projection Profilometry
FTP	Fourier Transform Profilometry
WFT	Windowed Fourier Transform Profilometry
WT	Wavelet Transform
PSP	Phase Shift Profilometry
SPU	Spatial Phase Unwrapping
TPU	Temporal Phase Unwrapping
MMP	Modulation Measuring Profilometry
DNNs	Deep Neural Networks
CNN	Convolutional Neural Network
PCCD	Precise Contour and Coarse Depth
GMDF	Global Multi-Dimensional Fusion
PDE	Progressive Depth Extractor
FSCE	Fringe Structure Consistency Evaluation
ViT	Vision Transformer
MHSA	Multi-Head Self-Attention
FFN	Feed-Forward Network
GeLU	Gaussian Error Linear Unit

## References

[1] Hu Y., Chen Q., Feng S., Zuo C. (2020). Microscopic fringe projection profilometry: A review. Opt. Lasers Eng..

[2] Huang L., Idir M., Zuo C., Asundi A. (2018). Review of phase measuring deflectometry. Opt. Lasers Eng..

[3] López-Alba E., Felipe-Sesé L., Schmeer S., A Díaz F. (2016). Optical low-cost and portable arrangement for full field 3D displacement measurement using a single camera. Meas. Sci. Technol..

[4] Zhang Z., Chang C., Liu X., Li Z., Shi Y., Gao N., Meng Z. (2021). Phase measuring deflectometry for obtaining 3D shape of specular surface: A review of the state-of-the-art. Opt. Eng..

[5] Jiang C., Jia S.H., Xu Y., Bao Q.C., Dong J., Lian Q. (2015). The application of multi-frequency fringe projection profilometry on the measurement of biological tissues. Bio-Med. Mater. Eng..

[6] Wu Y.X., Cai X.J., Zhu J.J., Yue H.M., Shao X.P. (2020). Analysis and reduction of the phase error caused by the non-impulse system psf in fringe projection profilometry. Opt. Lasers Eng..

[7] Xu J., Zhang S. (2020). Status, challenges, and future perspectives of fringe projection profilometry. Opt. Lasers Eng..

[8] Su X.Y., Chen W.J. (2001). Fourier transform profilometry: A review. Opt. Lasers Eng..

[9] Mao X.F., Su X.Y., Chen W.J., Jin H.L. (2010). A flexible calculation on improved Fourier transform profilometry. Optik.

[10] Zhang H.H., Zhang Q.C., Li Y., Liu Y.H. (2019). High Speed 3D Shape Measurement with Temporal Fourier Transform Profilometry. Appl. Sci..

[11] Kemao Q. (2007). Two-dimensional windowed Fourier transform for fringe pattern analysis: Principles, applications and implementations. Opt. Lasers Eng..

[12] Zhong J.G., Weng J.W. (2004). Spatial carrier-fringe pattern analysis by means of wavelet transform: Wavelet transform profilometry. Appl. Opt..

[13] Zhang S. (2018). Absolute phase retrieval methods for digital fringe projection profilometry: A review. Opt. Lasers Eng..

[14] Liu C.Y., Wang C.Y. (2020). Investigation of Phase Pattern Modulation for Digital Fringe Projection Profilometry. Meas. Sci. Rev..

[15] Wu Z.J., Guo W.B., Zhang Q.C. (2022). Two-frequency phase-shifting method vs. Gray-coded-based method in dynamic fringe projection profilometry: A comparative review. Opt. Lasers Eng..

[16] Zuo C., Feng S.J., Huang L., Tao T.Y., Yin W., Chen Q. (2018). Phase shifting algorithms for fringe projection profilometry: A review. Opt. Lasers Eng..

[17] Lu L., Suresh V., Zheng Y., Wang Y., Xi J., Li B. (2021). Motion induced error reduction methods for phase shifting profilometry: A review. Opt. Lasers Eng..

[18] Jiang H., Xu Y., Zhang C., Xu Z.-J., Huang J., Tan H., Lu J. (2020). An Algorithm Combining the Branch-Cut Method and Rhombus Phase Unwrapping Algorithm. J. Phys. Conf. Ser..

[19] Xu C., Cao Y.P., Wu H.T., Li H.M., Zhang H.C., An H.H. (2022). Curtain-type phase unwrapping algorithm. Opt. Eng..

[20] He X., Kemao Q. (2020). A comparison of n-ary simple code and n-ary gray code phase unwrapping in high-speed fringe projection profilometry. Opt. Lasers Eng..

[21] Zuo C., Huang L., Zhang M.L., Chen Q., Asundi A. (2016). Temporal phase unwrapping algorithms for fringe projection profilometry: A comparative review. Opt. Lasers Eng..

[22] Wang J., Cao Y.P., Wu H.T., Wei Z.M. (2023). Absolute phase retrieval based on fringe amplitude encoding without any additional auxiliary pattern. Opt. Express.

[23] Wei Z., Cao Y., Wu H., Xu C., Ruan G., Wu F., Li C. (2024). Dynamic phase-differencing profilometry with number-theoretical phase unwrapping and interleaved projection. Opt. Express.

[24] Zhong J.G., Zhang Y.L. (2001). Absolute phase-measurement technique based on number theory in multifrequency grating projection profilometry. Appl. Opt..

[25] An H.H., Cao Y.P., Zhang Y., Li H.M. (2023). Phase-Shifting Temporal Phase Unwrapping Algorithm for High-Speed Fringe Projection Profilometry. IEEE Trans. Instrum. Meas..

[26] Yin Z.Y., Du Y.F., She P.Y., He X.Y., Yang F.J. (2021). Generalized 2-step phase-shifting algorithm for fringe projection. Opt. Express.

[27] Wang Y.J., Zhang S. (2012). Novel phase-coding method for absolute phase retrieval. Opt. Lett..

[28] Lu M.T., Su X.Y., Cao Y.P., You Z.S., Zhong M. (2016). Modulation measuring profilometry with cross grating projection and single shot for dynamic 3D shape measurement. Opt. Lasers Eng..

[29] Li C.M., Cao Y.P., Chen C., Wan Y.Y., Fu G.K., Wang Y.P. (2017). Computer-generated Moire profilometry. Opt. Express.

[30] Dirckx J.J.J., Decraemer W.F., Dielis G. (1988). Phase-shift method based on object translation for full field automatic 3-D surface reconstruction from moire topograms. Appl. Opt..

[31] Eguchi A., Milster T.D. (2019). Single-shot phase retrieval with complex diversity. Opt. Lett..

[32] Gupta A.K., Mahendra R., Nishchal N.K. (2020). Single-shot phase imaging based on transport of intensity equation. Opt. Commun..

[33] He X.L., Liu C., Zhu J.Q. (2018). Single-shot phase retrieval based on axial phase diversity. Optik.

[34] Zhou H.Q., Li X., Ullah N., Geng G.Z., Li J.J., Li X.W., Wang Y.T., Huang L.L. (2022). Single-shot phase retrieval based on anisotropic metasurface. Appl. Phys. Lett..

[35] Spoorthi G.E., Gorthi S., Gorthi R.K.S.S. (2019). PhaseNet: A Deep Convolutional Neural Network for Two-Dimensional Phase Unwrapping. IEEE Signal Process. Lett..

[36] Spoorthi G.E., Gorthi R.K.S.S., Gorthi S. (2020). PhaseNet 2.0: Phase Unwrapping of Noisy Data Based on Deep Learning Approach. IEEE Trans. Image Process..

[37] Feng S.J., Chen Q., Gu G.H., Tao T.Y., Zhang L., Hu Y., Yin W., Zuo C. (2019). Fringe pattern analysis using deep learning. Adv. Photonics.

[38] Qian J.M., Feng S.J., Li Y.X., Tao T.Y., Han J., Chen Q., Zuo C. (2020). Single-shot absolute 3D shape measurement with deep-learning-based color fringe projection profilometry. Opt. Lett..

[39] Li Y.X., Qian J.M., Feng S.J., Chen Q., Zuo C. (2022). Composite fringe projection deep learning profilometry for single-shot absolute 3D shape measurement. Opt. Express.

[40] Qi Z.S., Liu X.J., Pang J.Q., Hao Y.F., Hu R., Zhang Y.N. (2023). PSNet: A Deep Learning Model-Based Single-Shot Digital Phase-Shifting Algorithm. Sensors.

[41] Wang L.L., Xue W.K., Wang C.Y., Gao Q., Liang W.J., Zhang Y.W. (2023). Depth estimation from a single-shot fringe pattern based on DD-Inceptionv2-UNet. Appl. Opt..

[42] Nguyen H., Tran T., Wang Y.Z., Wang Z.Y. (2021). Three-dimensional Shape Reconstruction from Single-shot Speckle Image Using Deep Convolutional Neural Networks. Opt. Lasers Eng..

[43] Nguyen H., Wang Y.Z., Wang Z.Y. (2020). Single-Shot 3D Shape Reconstruction Using Structured Light and Deep Convolutional Neural Networks. Sensors.

[44] Van der Jeught S., Dirckx J.J.J. (2019). Deep neural networks for single shot structured light profilometry. Opt. Express.

[45] Han D., Shin J., Kim N., Hwang S., Choi Y. (2022). TransDSSL: Transformer Based Depth Estimation via Self-Supervised Learning. IEEE Robot. Autom. Lett..

[46] Papa L., Russo P., Amerini I. (2023). METER: A Mobile Vision Transformer Architecture for Monocular Depth Estimation. IEEE Trans. Circuits Syst. Video Technol..

[47] Yan L., Yu F.Y., Dong C. (2023). EMTNet: Efficient mobile transformer network for real-time monocular depth estimation. Pattern Anal. Appl..

[48] Wang L., Lu D.Q., Tao J.Q., Qiu R.W. (2022). Single-shot structured light projection profilometry with SwinConvUNet. Opt. Eng..

[49] Zhu X.J., Han Z.Q., Zhang Z.Z., Song L.M., Wang H.Y., Guo Q.H. (2023). PCTNet: Depth estimation from single structured light image with a parallel CNN-transformer network. Meas. Sci. Technol..

[50] Dosovitskiy A., Beyer L., Kolesnikov A., Weissenborn D., Zhai X., Unterthiner T., Dehghani M., Minderer M., Heigold G., Gelly S. (2020). An Image is Worth 16x16 Words: Transformers for Image Recognition at Scale. arXiv.

[51] Choi S., Takahashi S., Sasaki O., Suzuki T. (2014). Three-dimensional step-height measurement using sinusoidal wavelength scanning interferometer with four-step phase-shift method. Opt. Eng..

